# Paradoxical expression of *IL-28B *mRNA in peripheral blood in human T-cell leukemia virus Type-1 mono-infection and co-infection with hepatitis C Virus

**DOI:** 10.1186/1743-422X-9-40

**Published:** 2012-02-15

**Authors:** Shimeru Kamihira, Tetsuya Usui, Tatsuki Ichikawa, Naoki Uno, Yoshitomo Morinaga, Sayaka Mori, Kazuhiro Nagai, Daisuke Sasaki, Hiroo Hasegawa, Katsunori Yanagihara, Takuya Honda, Yasuaki Yamada, Masako Iwanaga, Takashi Kanematu, Kazuhiko Nakao

**Affiliations:** 1 Department of Laboratory Medicine, Nagasaki University Graduate School of Biomedical Sciences, Nagasaki, 852-8501, Japan; 2 Central Diagnostic Laboratory of Nagasaki University Hospital, Nagasaki, 852-8501, Japan; 3 Department of Gastroenterology and Hepatology, Nagasaki University Graduate School of Biomedical Sciences, Nagasaki, 852-8501, Japan; 4 Faculty of Wellness Studies, Kwassui Women's University, Nagasaki, 850-8515, Japan; 5 Division of Surgical Oncology, Department of Translational Medical Science, Nagasaki University Graduate School of Biomedical Sciences, Nagasaki, 852-8501, Japan

**Keywords:** IL-28B, IL-λ3, HTLV-1, HCV, SNP

## Abstract

**Background:**

Human T-cell leukemia virus type-1 (HTLV-1) carriers co-infected with and hepatitis C virus (HCV) have been known to be at higher risk of their related diseases than mono-infected individuals. The recent studies clarified that IL-28B polymorphism rs8099917 is associated with not only the HCV therapeutic response by IFN, but also innate immunity and antiviral activity. The aim of our research was to clarify study whether IL-28B gene polymorphism (rs8099917) is associated with HTLV-1/HCV co-infection.

**Results:**

The genotyping and viral-serological analysis for 340 individuals showed that IL-28B genotype distribution of rs8099917 SNP did not differ significantly by respective viral infection status. However, the IL-28B mRNA expression level was 3.8 fold higher in HTLV-1 mono-infection than HTLV-1/HCV co-infection. The high expression level was associated with TT (OR, 6.25), whiles the low expression was associated with co-infection of the two viruses (OR, 9.5). However, there was no association between down-regulation and ATL development (OR, 0.8).

**Conclusion:**

HTLV-1 mono-infection up-regulates the expression of IL-28B transcripts in genotype-dependent manner, whiles HTLV-1/HCV co-infection down-regulates regardless of ATL development.

## Introduction

A retrovirus, human T-cell leukemia virus type-1 (HTLV-1), and a positive-strand RNA virus, hepatitis C virus (HCV), are completely different in terms of virologic characteristics. Nevertheless, they play a similar role in the pathogenesis of viral-induced malignant neoplasms, such as adult T-cell leukemia (ATL) in HTLV-1- infected individuals, and hepatocellular carcinoma (HCC) and B-cell lymphoma in HCV- infected individuals, during long-term chronic infections.

Furthermore, it is known that co-infection with HCV and HTLV-1 is frequently observed in an area endemic for HTLV-1. HCV/HTLV-1 co-infected individuals have been reported to be at higher risk for developing HCC than those infected with HCV alone [1-3]. Although the pathologic mechanism of the co-infection remains to be elucidated, it is thought that the impaired immunity due to HTLV-1 infection may contribute to HCV infection and HCV-related disorders, which is suggested by previous reports. Kohno et al. reported that the severe immunodeficiency and anergic state in patients with ATL may be associated with a functional property of leukemic cells originating from regulatory T-cells expressing CD4, CD25, CCR4, GITR and Foxp3 [4]. Kishihara et al. also reported that impairment of the immune response by HTLV-1 could explain the reduced effectiveness of interferon (IFN) treatment in patients co-infected with HTLV-1 and HCV [5].

Recently, genome-wide association studies of patients with HCV have made great advances in viral clearance associated with *IL-28B *single nucleotide polymorphisms (SNP) [6,7]. IL-28B is a type III Lambda interferon (IFN-λ) and a cytokine similar to IL-10 with IFN-like activities [8]. This new IFN-λ family includes IFN-λ1 (IL-29), IFN-λ2 (IL-28A) and IFN-λ3 (IL-28B) [9]. Although the IFN-λ genomic structure resembles that of the IL-10 family [10], the amino acid and functional level of IFN-λs are more closely related to type I IFNs than IL-10. The IFN-λs are induced by stimulation with several single-strand RNAs (ssRNA) and several kinds of viruses. The IL-28B SNPs, such as rs8099917, rs12979860, and 12980275, have been reported to be associated with spontaneous clearance [10], innate HCV immunity [9], HCV-related disease chronicity, and therapeutic response to pegIFN-α and ribavirin (RBV) [6,7].

From these observations, we hypothesized that IFN-λ3 encoded from the *IL-28B *gene would be associated with HTLV-1 infection. The aim of the present study was to examine the mutual association between IL-28B polymorphism (rs8099917 SNP) and mono-infected-HTLV-1 and co-infected HTLV-1 with HCV subjects.

## Materials and methods

### Clinical subjects

All subjects were of Japanese origin living in Nagasaki City, an endemic area for HTLV-1 in Japan. For genomic specimens, 340 blood samples were randomly collected from patients who visited a liver clinic and liver transplantation center from April 2009 to March 2011 from the departments of Hepatology and a Hematology Clinic. One hundred and twenty-four of the 340 samples were also available for total RNA tests. Accordingly, most patients had either chronic liver disease (CLD) or adult T-cell leukemia (ATL). This study was done under informed consent after the approval of the Nagasaki University hospital IRB (IRB Approval No.10050). Since the samples used here were un-linked materials, patient information was restricted.

### Cell lines

Eight HTLV-1-infected T-cell lines, Hut 102, MT-1, MT-2, ST1, KK1, KOB, OMT, and LMY-1, were used for IL-28B mRNA quantification. The first three were purchased and latter five were established in our laboratory [11].

### Serological and genetic tests for HCV and HTLV-1

HCV and HTLV-1 infections were mainly serologically detected using commercially available kits, CLEIA-anti-HTLV-1, Lumipulse-II Ortho HCV (Fujirebio-INC, Tokyo, Japan). The confirming examination was genetically performed by the Cobas TaqMan HCV test (TaqMan HCV; Roche Tokyo INC, Tokyo, Japan) for HCV and in-house HTLV-1 proviral real-time RT quantifiable PCR [12]. Genomic DNA and total RNA were extracted from peripheral blood mononuclear cells (PBMC) using commercially available QuickGene DNA Whole blood kits (FUJIFILM Corp., Tokyo, Japan) and PureLink RNA Micro Kits (Invitrogen Corp., Carlsbad, Ca, USA). The extraction protocol was performed according to the manufacturer's instructions.

### Genotyping for SNPs

SNP genotyping was performed using multiplex PCR amplification and Pyrosequencing technology. To amplify target regions, newly designed biotinylated-primers were employed: sense and anti-sense for rs8099917, 5'-TCCTCCTTTTGTTTTCCTTTCTG-3' and 5'-AAAAAGCCAGCTACCAAACTGT-3'. Then, the amplicon was sequenced according to the manufacturer's instructions based on Pyrosequence technology (Qiagen, Hilden, Germany). Biotin-labeled amplicons from the 1st PCR were captured by binding to streptavidin-coated Sepharose beads, and DNA was denatured to produce an ssDNA template for the Pyrosequencing assay. The ssDNA was released and combined with the sequencing primer, which was extended during the Pyrosequencing reaction to provide the sequence of the template DNA. Pyrosequencing data were produced in the form of Pyrograms, and genotypes were assigned by the peak pattern presented in the Pyrogram.

### Real-time reverse transcription (RT) quantifiable PCR for IL-28B mRNA

mRNA for IL-28B transcribed into cDNA by a GoScript^™ ^RT System (Promega, Madison, WI, USA) was quantified by a LightCycler System (Rosche, Mannheim, Germany) using newly designed sense and anti-sense primers, 5'-AAGGACTGCAAGTGCCGCT-3' and 5'-GCTGGTCCAAGACATCCC-3' (AY129149). A standard curve was generated using a tenfold dilution method with a reference material derived from pTAC-1.2735 inserted with 166 base fragments including the target. The amplicon was assayed by the Cyber green method. The raw data were normalized by *abl *mRNA density and evaluated as the relative value for *abl *gene expression calculated by *IL-28B *data/*abl *data × 10^4^, modified from our previous mRNA real time RT qPCR method [12].

### Statistical analysis

The minor-allele frequency (MiAF) was set as the less frequent allele in a population for SNPs analyzed. Viral infectious status was divided into 4 groups of HTLV-1 mono-infection, HCV mono-infection, HTLV-1/HCV-co-infection, and non-infection (double negative; DN). Differences in the genotype distribution of IL-28B SNPs among groups were compared using the Chi-square or Fisher exact test. The level of mRNA expression was compared using the Mann Whitney U test. Correlation analysis was performed by the Nonparametric Spearman's rank correlation method. The relationship between a factor and an outcome was estimated the magnitude of the association by the odds ratio with 95% confidence intervals (95%CI). Statistical analysis was performed using SAS 9.1. The statistical significance level was set at 0.05.

## Results

### IL-28B genotypes and the sero-status

Three hundred and forty samples were genotyped on IL-28B rs8099917 SNP and were serologically examined for viral infection of HTLV-1 and HCV. As summarized in Table 1. They consisted of 263 (77.4%) major TT homozygotes, 171 (20.9%) minor TG heterozygotes, and 6 (1.8%) minor GG homozygotes. The virus tests revealed that 59 were negative for both HTLV-1 and HCV, 73 were positive for HTLV-1 alone, 179 were positive for HCV alone and 29 were positive for both viruses. The genotypic distributions, as well as minor allele frequency (MAF) of the IL-28B gene, did not significantly differ among each viral infection status as a control of no-infection.

**Table 1:** IL-28B genetic distribution and allele frequency in stratification based on the combination of HTLV-1 and HCV infection

	Genotype r(rs8099917)				Allele fequency		
	**No**	**TT**	**TG**	**GG**	**T**	**G**	

All cases	340	263(77.4%)	71(20.9%)	6(1.8%)	0.86	0.14	

1) non-Infection	59	45(76.3)	10(16.9)	4(6.8)	0.84	0.15	

2) HTLV-1 mono	73	55(75.3)	17(23.3)	1(1.9)	0.87	0.13	*P *= 0.90

ATL patients carriers	47	37(78.7)	10(21.3)	0(0.0)	0.89	0.11	*P *= 0.11
	
	26	18(69.2)	7(26.9)	1(3.8)	0.82	0.18	*P *= 0.46

3) HCV-mmono	179	141(78.7)	37(20.7)	1(1.0)	0.89	0.11	*P *= 0.68

4) co-infection	29	22(75.9)	7(24.1)	0(0.0)	0.88	0.14	*P *= 0.9

There was no significant difference in the genetic distribution and allele frequency among respective infectious states

*P *values were compared with non infection

Since the HTLV-1 mono-infection group consisted of 47 ATL patients and 26 HTLV-1 carriers, we stratified the two groups of ATL patients and carriers and the minor allele frequencies of the two groups were compared; the difference between that of ATL and carriers was not statistically significant (*p *= 0.21). The prevalence of TT was not different statistically either (*p *= .495).

Next, the expression levels of IL-28B were quantified using 124 samples randomly collected during this study period.

### IL-28B mRNA expression level and HCV/HTLV-1 co-infection

**Figure 1:**
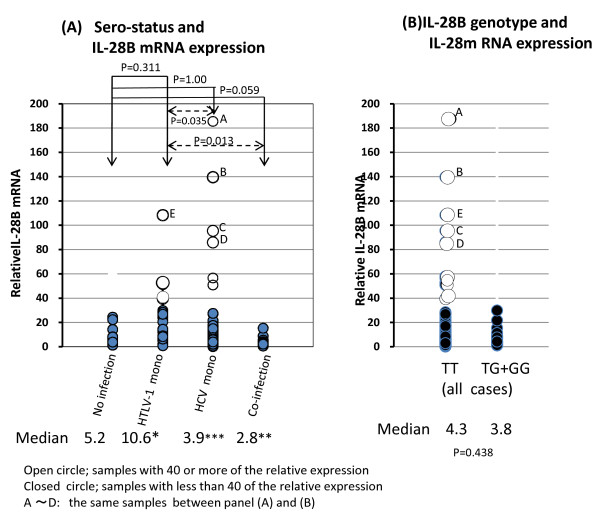
**Relative individual IL-28B mRNA levels in peripheral blood mononuclear cells among viral infectious groups (a) and among the TT and TG/GG genotypes (b)**. The median values were significantly different between * vs ** and *** vs **. Open circles; those with high IL28BmRNA with TT homozygotes.

**Figure 2:**
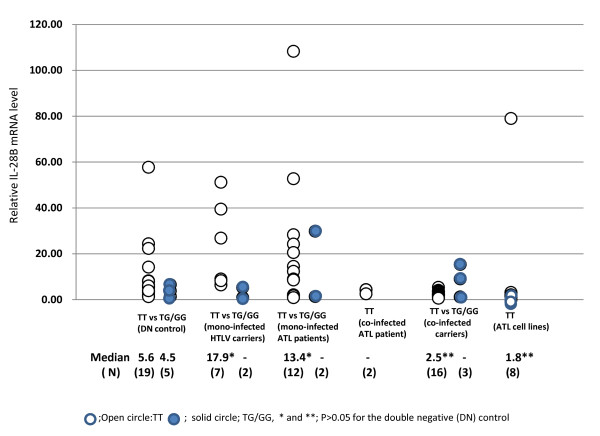
**Individual relative expression density of IL-28B mRNA in TT vs TG/GG derived from 24 double-negative (DN) controls consisting of 19TT and 5TG/GG, 9 mono- infected HTLV-1 carriers, 14 mono-infected and 2 co-infected ATL patients, 19 co-infected carriers and 8 ATL cell lines samples**.

The expression levels of IL-28B mRNA were generally low in most cases with a median value of 5.2 in no-infection, 10.6 in HTLV-1 mono-infection, 3.9 in HCV mono-infection, and 2.8 in HTLV-1/HCV co-infection (Figure 1a). Notably, a small number of measurement values shown as open circles was high, and they were distributed only within the HTLV-1 mono-infected and HCV mono-infected groups. Moreover, all of those who had high values were exclusively TT homozygous, as shown in Figure 1b (samples marked by ^A-E) ^were the same in Figure 1(a) and Figure 1(b)). Surprisingly, the median value was the highest in HTLV-1 mono-infection and the lowest in the co-infection group (10.6 versus 2.8; *p *= 0.013). Therefore, to clarify whether ATL cells directly affect the expression of IL-28B mRNA, we compared the mRNA expression levels in mainly HTLV-1 carriers, ATL patients with ATL cells, and ATL cell lines. As shown in Figure 2, the median values were significantly higher in mono HTLV-1 carriers with TT (17.9 vs 5.6, *P *< 0.05) and ATL patients with TT having ATL cells than those of non-infected individuals (13.4 vs 5.6, *p *< 0.05). No high expression level was observed in two ATL or 16 carriers with HTLV-1/HCV co-infection. Surprisingly, these data were lower rather those from TG/GG. On the other hand, IL-28B mRNA expression in 8 HTLV-1-infected T-cell lines was undetectable in all but one (Hut 102). The genotype was TT in all cell lines.

**Figure 3:**
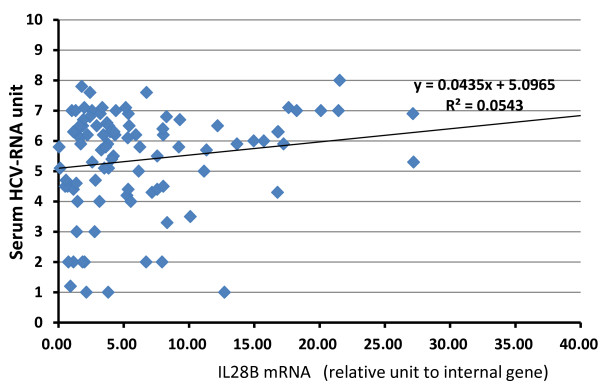
**Correlation between serum HCV-RNA level and IL-28B mRNA**. No-correlation (r^2^) = 0.0543.

In addition, there was no correlation between the IL-28B mRNA levels and HCV-RNA levels (non-parametric Spearman's rank correlation, R ^2 ^= 0.0543, Figure 3).

### Assessment by odds ratio analysis for an outcome if a risk factor is present

As shown in Figure 2, HTLV-1 was revealed to have an association similar to HCV and IL-28B mRNA. However, the up-regulated-action of HTLV-1 was nullified if the virus was co-infected with HCV. The prevalence of a major TT and minor TG/GG was similar among individuals infected with either HTLV-1 or HCV, as well as the allele frequency, indicating that there is no specific correlation between IL-28B and HTLV-1. Thus, to approach a causative clue, assessment by odds ratio (OR) analysis was performed (Table 2). Only the high mRNA level besides 3 states of HTLV-1 mono-infection, co-infection with HCV and ATL was associated with TT genotype (OR = 6.25). On the other hand, down-regulation of the mRNA density was defined as HTLV-1/HCV co-infection (OR = 9.5 *p *= 0.004), but low expression was not associated with ATL development (OR = 0.8, *p *= 0.81).

**Table 2:** Assessment by odds ratio analysis for an outcome if a risk factor is present

	factor			
**(A) Outcome**	**dependent independent**	**Odds ratio**	**95%CI**	**P**

1) HTLV-1 mono-infection	TT vs TG/GG	1.11	0.62-1.99	0.72

2) Co-infection	TT vs TG/GG	0.54	0.04-6.88	1.00

3) High mRNA Expression*	TT vs TG/GG	6.25	1.16-33.75	0.04

4) ATL (B)	TT vs TG/GG	1.50	0.60-3.75	0.39

5) Low mRNA Expression	HTLV-1 mono vs DN	0.34	0.06-2.04	0.24

6) Low mRNA Expression	HCV mono vs DN	0.29	0.07-2.23	0.15

7)) Low mRNA Expression	Co-Inf** vs HTLV-1-mono**	9.5	2.06-43.76	0.004

8) ATL	low expression or not	0.8	0.14-4.74	0.81

(A) Upper 4 lines; assessing the risk of 1) HTLV-1 persistent infection, 2) super-imposed HTLV-1 infection with HCV (co-infection), 3) high IL-28B mRNA expression, and 4) ATL development when the genotype is a risk factor (B) Lower 4 lines; assessing the risk factors described in the outcome, the IL-28B mRNA expression level in peripheral blood (5, 6, and 7), and ATL development (8). Consequently, similarly to HCV, HTLV-1 is associated with up-regulation of IL-28B mRNA along with the TT homozygote, and co-infection with HTLV-1 and HCV paradoxically down-regulates the mRNA level

*; IL-28B Expression level, Co-inf = co-infection with HTLV-1 and HCV, mono = mono-infection

## Discussion

Although co-infection with HTLV-1 and HCV has been shown to result in higher rates of cirrhosis and increased death from liver diseases [1,2], the caustic mechanism by which the co-infection affects HCV pathogenesis remains to be elucidated. Some clue to the mechanism may be found by studying the relation between IL-28B genotypes and co-infection, because IL-28B encoding IFN-λs are categorized as type 3 IFNs and are potent endogenous anti-viral cytokines. They signal via JAK/STAT intracellular pathways and up-regulated transcription of IFN-stimulated genes (ISGs) that are required to control viral infection [13]. Here, we investigated whether IL-28B polymorphism rs8099917 is associated with co-infection status.

The present study is the first to reveal that the IL-28B genotype is not associated with stratification based on the combination of HTLV-1 and HCV infection; no infection for both (double negative; DN), HTLV-1 mono-infection, HCV mono-infection and HTLV-1/HCV co-infection. Similarly, the frequency of the major TT homozygotes was not associated among ATL patients and HTLV-1 carriers (Table 2). These two findings suggest that the SNP rs8099917 is not associated with susceptibility to HTLV-1 infection or the development of ATL. On the other hand, all of ATL cell- or HTLV-1-infected T-cell- lines examined were exclusively TT homozygous, implying that HTLV-1-infected cells carrying TT homozygotes may immortalize easily in vitro.

Next, we found a strange phenomenon that the IL-28B mRNA expression levels in peripheral blood were lower in samples with HTLV-1/HCV co-infection than in samples with either HTLV-1 or HCV alone, especially significantly for HTLV-1 mono-infection. In particular, samples carrying TT homozygotes were strongly down-regulated, more than the minor TG hetero- and GG-homozygotes. Why are the mRNA expression levels different in mono- and dual-infection? Although it is not known how rs8099917 affects the action of IL-28B, presumably it alters the immune function to viruses. In addition to a common anti-viral IFN-stimulating signal pathway, HTLV-1 may use an alternative anti-viral pathway like HBV [14], because the HTLV-1 provirus is integrated into host genomic DNA and replicates in distinctive life cycle kinetics. Moreover, ATL originates from Treg cells, which play a central role in suppressing immunity [15]. However, this cannot fully explain the impairment in the HTLV-1 carrier's immunity because no ATL cells are present during the carrier period. Thus, we noted IFN-λ(IL-29, IL-28), which was recently discovered as a type III IFNs with anti-virus ability, anti-tumor and immune responses [16-18].

From our results, the IL-28B expression level was higher in HTLV-1 mono-infected individuals including ATL patients. IFN-λis usually up-regulated through activation of the NF-kappaB pathway after viral infection. Actually, the Sendai virus, an influenza A virus, and others have been demonstrated to activate the NF-kappa B pathway, resulting in up-regulated IL-28B expression [19,20]. Accordingly, the highest up-regulation of IFN-λ3 in HTLV-1 mono-infection may be explained by virtue of a viral protein of HTLV-1 having strong NF-kB activating ability. Moreover, it is instructive that IFN-λhas a potent function to expand Treg cells [21], which are mainly infected with HTLV-1, predisposing development of ATL. However, there has not yet been evidence that co-infection with HCV damages Tax action.

Of IL-28B producing cells in the literature, most cells in the blood are described as having a weak or absent expression under the steady state conditions. Li et al. [9] reported that IL-28B mRNA is not always expressed in virally infected cells. Actually, our findings in HTLV-1-infected cases also showed that at least the main producing cells are likely to be cells other than ATL cells because most cell lines from ATL and some blood samples containing ATL cells were expressed faintly. At present, plasmacytoid dendritic cells are indicated to be the most potent producers of IFN-λs [19]. On the other hand, IFN-λ3 reportedly has the functions of proliferating Treg cells which are the origin of ATL cells, suggesting that HTLV-1 is associated with up-regulation via Treg cells infected with HTLV-1.

In conclusion, we found an unusual phenomenon in that the expression of IL-28B mRNA was affected by not only the IL-28B rs8099917 genotype, but also co-infected HTLV-1 with HCV. This will contribute to a better understanding the enigmatic impairment of immunity in the HTLV-1 carrier state, including co-infection with HTLV-1 and HCV.

## Abbreviations

HTLV-1: Human T-cell leukemia virus type -1; HCV: Hepatitis C virus; SNP: Single nucleotide polymorphism; IFN: Interferon; PBMC: Peripheral blood mononuclear cell; PCR: Polymerase chain reaction; MAF: Minor-allele frequency.

## Competing interests

The authors declare that they have no competing interests.

## Authors' contributions

SK designed the study and wrote the manuscript, and SM, TU, KN, DS HH, KY, NU, YM analyzed the genotype, TK collected samples, and TK, KN, MI and SK organized and assessed the data. All authors interpreted the data and were financially supported. All authors read and approved the final manuscript.
